# Clinician and administrator perspectives on outpatient administration of ciltacabtagene autoleucel in relapsed or refractory multiple myeloma

**DOI:** 10.3389/fimmu.2024.1405452

**Published:** 2024-06-10

**Authors:** Doris K. Hansen, Binod Dhakal, Mehdi Hamadani, David Dingli, Tania Jain, Carol Ann Huff, Murali Janakiram, Yi-Hsuan Liu, Kevin C. De Braganca, Nicole Lodowski, Jennifer Sander, Peter Okorozo, Lindsay McFarland, Matthew Perciavalle, Stephen Huo, Zaina P. Qureshi, Krina K. Patel

**Affiliations:** ^1^Department of Blood and Marrow Transplant and Cellular Immunotherapy, H. Lee Moffitt Cancer Center and Research Institute, Tampa, FL, United States; ^2^Blood and Marrow Transplant (BMT) and Cellular Therapy, Division of Hematology and Oncology, Medical College of Wisconsin, Milwaukee, WI, United States; ^3^Division of Hematology, Mayo Clinic, Rochester, MN, United States; ^4^Division of Hematological Malignancies and Bone Marrow Transplantation, The Sidney Kimmel Comprehensive Cancer Center, Johns Hopkins Hospital, Baltimore, MD, United States; ^5^Department of Hematology and Hematopoietic Cell Transplantation, City of Hope, Duarte, CA, United States; ^6^Real-World Value & Evidence, Janssen Scientific Affairs, LLC, a Johnson & Johnson company, Horsham, PA, United States; ^7^Medical Affairs, Janssen Research & Development, LLC, a Johnson & Johnson company, Raritan, NJ, United States; ^8^Avalere Health, New York, NY, United States; ^9^Legend Biotech USA Inc., Somerset, NJ, United States; ^10^Department of Lymphoma and Myeloma, The University of Texas MD Anderson Cancer Center, Houston, TX, United States

**Keywords:** ciltacabtagene autoleucel, cilta-cel, outpatient, car t therapy, CAR T-cell, relapsed or refractory multiple myeloma, multiple myeloma, ambulatory care

## Abstract

**Introduction:**

Chimeric antigen receptor (CAR) T-cell therapy (CAR T therapy) is a treatment option for patients with relapsed or refractory multiple myeloma that has led to unprecedented treatment outcomes. Among CAR T therapies available, ciltacabtagene autoleucel (cilta-cel) is a good candidate for outpatient administration due to its generally predictable safety profile. There are multiple advantages of outpatient administration of cilta-cel, including reduced healthcare burden, expanded access, and patient autonomy. This mixed methods qualitative study aimed to identify key factors for outpatient administration of CAR T and best practice recommendations by combining a targeted literature review with expert interviews and panels.

**Methods:**

The targeted review (Phase 1) aimed to identify factors for outpatient CAR T administration in the US and determine key topics for the exploratory interviews (Phase 2) and expert panels (Phase 3), which aimed to inform on best practices and challenges of outpatient CAR T administration (focusing on cilta-cel). Participants in clinical and administrative positions based in treatment centers that had experience with real-world outpatient administration of cilta-cel were recruited.

**Results:**

Seventeen studies were identified in Phase 1. Key factors for outpatient administration included the development of protocols for CAR T complications, education for caregivers, outpatient specialists, hospital staff, and emergency services staff for identification and referral after possible adverse events, the creation of multidisciplinary teams for effective communication and management, straightforward patient intake processes encompassing financial eligibility review and provision of patient education materials, and close patient monitoring throughout the treatment journey. In Phase 2, 5 participants from 2 centers were interviewed. In Phase 3, 14 participants across 6 treatment centers were interviewed. Two 90-minute virtual panel discussions took place. All participants agreed that cilta-cel can be safely and effectively administered in an outpatient setting. Key recommendations included the creation of educational resources for patients and caregivers, the development of standard operating procedures, dedicated outpatient infrastructure and establishment of interdisciplinary teams, outpatient monitoring for toxicity management, and monitoring of the reimbursement landscape.

**Discussion:**

This study offers a comprehensive understanding of the feasibility of outpatient cilta-cel administration in participating CAR T centers and provides actionable recommendations while acknowledging existing challenges.

## Introduction

Chimeric antigen receptor (CAR) T-cell therapy (CAR T therapy) has transformed treatment paradigms for patients with B-cell malignancies, including leukemia, lymphoma, and multiple myeloma (1, 2). CAR T cell therapy involves genetically modifying a patient’s own T-cells *ex-vivo* to express a CAR, generating specificity of the patients’ T-cells to specific proteins on tumor cells, and has led to a remarkable improvement in treatment outcomes such as overall response and overall survival (1, 3, 4).

Notably, ciltacabtagene autoleucel (cilta-cel) was approved in the US for use in adults with relapsed/refractory multiple myeloma (RRMM) after 4 or more prior lines of therapy in February 2022 based on the results of the pivotal CARTITUDE-1 trial (5). Historically, most CAR Ts were administered in the inpatient setting due to the rapid onset of adverse events (AEs) associated with immune-mediated toxicities, including cytokine release syndrome (CRS) and immune effector cell-associated neurotoxicity syndrome (ICANS) (6). Of the CAR Ts that are commercially available, cilta-cel is often perceived to be a suitable candidate for administration in an outpatient setting due to its generally predictable immune-mediated toxicity profile, including delayed onset of CRS and ICANS compared with other CAR Ts (5, 7–12). Specifically, CRS onset usually occurs between 7 and 8 days after cilta-cel infusion (5, 11), instead of between 1 to 3 days after infusion for other CAR Ts, such as idecabtagene vicleucel (ide-cel) or axicabtagene ciloleucel (axi-cel) (7–10, 12, 13). Similarly, onset of ICANS after cilta-cel has been reported at a median of 7 days, versus 2 and 3 days for ide-cel and tisagenlecleucel (tisa-cel), respectively (12). While patients in CARTITUDE-1 received cilta-cel exclusively in an inpatient setting (5), robust infrastructure planning at some centers across the United States (US) has fostered outpatient administration of commercially available cilta-cel (14–16). In the subsequent CARTITUDE-2 trial, 2 patients successfully received outpatient infusion of cilta-cel, demonstrating a generally manageable safety profile in this setting (17, 18).

There are multiple advantages of outpatient administration of cilta-cel, including reduced healthcare burden and expanded treatment access for patients (19, 20). A cost per responder analysis from the CARTITUDE-4 clinical trial population estimated a lower cost per complete responder and cost per month in progression-free survival (PFS) for cilta-cel in a population where 30% of patients received outpatient CAR T infusion compared to a full inpatient CAR T population (savings of $7,598 per complete responder and $294 per month in PFS) (20). In addition, availability of outpatient services may better align with patient preference for a quicker return to a normal routine (6) and improved overall access to novel therapeutic options (19). Research also suggests that patients’ quality of life is generally better in outpatient versus inpatient settings (21), due to increased participation in social activities, avoided hospital-acquired infections, and reduced financial stress (21–23).

As real-world experience with CAR T increases, administration in the outpatient setting is expanding (14–16, 24), particularly for CAR Ts that are associated with AEs that potentially occur several days after infusion, such as cilta-cel. The objective of this mixed methods qualitative study was to evaluate clinical and administrative perspectives on outpatient administration of commercial cilta-cel to understand best practices from certified CAR T centers in the US. This will offer valuable insights to guide both new and established CAR T centers in the US to evaluate the feasibility of outpatient delivery of cilta-cel, encompassing both patient care and administrative considerations.

## Materials and methods

### Overview of study design and phases

A US-based mixed methods qualitative study was conducted from February 2022 to June 2023, using a 3-phase approach (**Table 1**).

**Table 1:** Phases of the qualitative study investigating outpatient administration of CAR T.

Study Phases	Objectives
Phase 1: Targeted literature review	• Identify factors for outpatient CAR T administration for RRMM in the US • Establish an understanding of key topics to inform subsequent phases
Phase 2: Two semi-structured interviews (60 minutes) with 2 cilta-cel outpatient centers (total n=5)	• Identify critical topics and questions for discussion with expert advisor panels on the outpatient administration of CAR T, with a focus on cilta-cel
Phase 3: Two expert panels (90 minutes) with 6 US-based cilta-cel outpatient centers (total n=14)	• Inform best practices for outpatient CAR T administration, with a focus on cilta-cel • Define approaches for establishing outpatient administration centers • Identify challenges and successes in real-world outpatient administration • Explore recommendations and areas of improvement for outpatient administration

CAR T, chimeric antigen receptor T-cell therapy; cilta-cel, ciltacabtagene autoleucel; RRMM, relapsed or refractory multiple myeloma; US, United States.

### Phase 1: targeted literature review

The objective of Phase 1 was to conduct a targeted literature review to identify factors for outpatient CAR T administration for RRMM in the US (e.g., best practices, challenges, differences between centers, ways to improve care coordination) and identify key topics to inform subsequent phases. Peer-reviewed publications, white papers, grey literature, conference abstracts, posters, and presentations published between 1 March 2017 – 1 March 2022 were identified using PubMed, as well as conference proceedings and congress websites from relevant professional organizations (including the American Society of Hematology, American Society of Clinical Oncology, and the American Society for Transplantation and Cellular Therapy). The following search terms were used: “outpatient CAR-T treatment,” “outpatient chimeric antigen receptor T-cell therapy,” “outpatient treatment CAR-T,” “outpatient administration CAR-T,” “CAR-T logistics,” “CAR-T logistical challenges,” and “CAR-T outpatient considerations”. Articles were excluded if they only detailed CAR T inpatient administration or if they were based on findings outside of the US.

Identified articles were reviewed and assessed for relevance by 2 researchers, and key findings were evaluated thematically across the patient journey through inductive/reflexive thematic and content analysis.

### Phase 2: semi-structured exploratory interviews

The objective of Phase 2 was to expand on insights obtained from Phase 1 by conducting semi-structured exploratory interviews to collect real-world information on the administration of CAR T, with a focus on cilta-cel. Critical topics discussed included best practices, challenges encountered, differences in barriers across patient populations (based on demographics) and institution settings (i.e., size of institution and location), and ways to improve care coordination and reimbursement. These topics informed questions for discussion during expert panel interviews (Phase 3).

First, treatment centers were identified through the CARVYKTI^®^ Certified Treatment Center locator (25). These included treatment centers across different geographical areas in the US with expertise and high volume of experience with commercial outpatient administration of cilta-cel (i.e., 10–15 patients, averaging to about 1 patient per month since FDA approval).

Panel participants with clinical and administrative (i.e., program director and nurse coordinators) positions were selected based on the following criteria: (1) treats or assists in managing adult patients with RRMM in the US; (2) based in a certified facility that has the ability to conduct outpatient administration of RRMM CAR Ts, including cilta-cel; (3) has experience administering cilta-cel, including at least one patient in the outpatient setting; (4) is a member of a CAR T care team, including hematologists/oncologists and CAR T administrators at the practice sites. Participants were excluded based on scheduling, institutional conflicts, or lack of commercial experience with outpatient cilta-cel administration. Participants in the study were unblinded to the identity of the study sponsor, and the study sponsor was unblinded as to the identity of the advisors.

Sixty-minute semi-structured interviews were conducted separately at each center in April and May 2023 via Microsoft Teams video conferencing. Prior to the interviews, pre-reading material was sent to participants to familiarize them with key topics based on the literature review conducted in Phase 1 (e.g., CAR T patient journey, patient identification and coordination, cilta-cel administration and patient monitoring, overall challenges, and best practices; see **Supplementary Table 1**). A discussion guide was developed to cover topics following themes identified in Phase 1, with probing questions designed to identify differentiating points in outpatient cilta-cel administration. Study investigators reviewed transcripts and assessed findings, with the aim of building upon results from Phase 1 and informing the discussion flow for Phase 3.

### Phase 3: expert panel interviews

The objective of Phase 3 was to inform on best practices for outpatient CAR T administration (with a focus on cilta-cel), including defining approaches for establishing outpatient centers, identifying challenges and successes in the real-world, and exploring recommendations and areas of improvement.

Six centers (14 experts) participated in the expert panel interviews, including the 2 centers from Phase 2 and 4 additional certified cilta-cel centers from varied geographical areas (i.e., South, West, Midwest, Mid-Atlantic regions of the US; **Table 2**). The identification of additional centers and participants followed the same methodology applied during Phase 2, including recruitment from US centers with known and applicable experience with commercial outpatient administration of cilta-cel. Two expert panel interviews with 7 experts per interview (with clinical and administrative representation in both interviews) were scheduled over 90 minutes using Microsoft Teams, with similar pre-reading material shared prior to panel initiation.

**Table 2:** Characteristics of centers that participated in the expert interviews and panels (Phase 2 and 3).

Outpatient Centers	Number of Participants in Expert Interviews (Phase 2)	Number of Participants in Expert Panels (Phase 3)	Location	Number of Cilta-Cel Outpatients Treated at Center	PPS-Exempt	Standalone Facility
Center A	n=2 1 treating clinician 1 CAR T nurse coordinator	n=3 1 treating clinician 1 CAR T nurse coordinator 1 CAR T administrator*	South	>30	Yes	Yes
Center B	n=3 1 director of BMT/CAR T program 1 treating clinician 1 CAR T program nurse coordinator	n=3 1 director of BMT/CAR T program 1 treating clinician 1 CAR T program nurse coordinator	Midwest	>15	No	No
Center C	–	n=2 1 treating clinician 1 CAR T program manager	Midwest	>15	No	No
Center D	–	n=2 1 treating clinician 1 CAR T program administrator	West	>15	Yes	Yes
Center E	–	n=2 1 treating clinician 1 CAR T coordinator	South	<15	Yes	No
Center F	–	n=2 1 cell therapy director 1 medical director	Mid-Atlantic	<15	No	No

*Participant included in Phase 3 only.

BMT, bone marrow transplant; CAR T, chimeric antigen receptor T-cell therapy; PPS, prospective payer system.

During the panels, experts were asked to rank various aspects of the decision-making processes for outpatient CAR T administration (i.e., institution space, reimbursement/incentives, physician/patient preferences, literature review, and expert panel perception) as of “low”, “moderate”, or “high” importance. In addition, experts provided feedback on the patient journey, identified important criteria for selecting patients who may receive cilta-cel in an outpatient setting, discussed insurance requirements, and provided recommendations for establishing outpatient CAR T administration centers. The panel transcripts were reviewed and summarized by study investigators to provide key takeaways and recommendations for the successful administration of outpatient CAR T, focusing on cilta-cel.

## Results

### Phase 1: targeted literature review

Seventeen articles describing the challenges and best practices for outpatient CAR T administration were identified in the targeted literature review (6, 26–41). Of note, at the time of the review in March 2022, there was no published literature specific to RRMM CAR T products administered in the outpatient setting.

According to the targeted literature review, outpatient CAR T can be successfully implemented by treatment centers with experienced clinical and administrative teams. Key drivers for the transition from inpatient to outpatient CAR T administration include patient preference, reduced healthcare resource utilization (including shorter length of stay), and toxicity management (6, 34).

An important element identified for successful transition of CAR T administration to the outpatient setting is the need for protocols for CAR T complications requiring emergency care, manufacturing delays, or unavailable resources (i.e., staff or patient room in hospitals) (6, 28). Logistical challenges include accurate prediction of manufacturing limitations and capacity through communication with manufacturing facilities (6, 30), as well as coordination of staffing (28) and 24-hour operation of emergency call systems for AE management (6). In addition, a well-developed and dedicated physical space (such as rooms and beds) in hospitals is a suggested best practice to facilitate the transition to inpatient management of severe AEs (28, 32).

Furthermore, education for caregivers, outpatient specialists, hospital staff, and emergency services staff for identification and referral of patients with potential AEs to the right facilities was identified as an important element of CAR T outpatient administration. Patient and caregiver education may include information on treatment plans and processes involved in therapy. This information may be delivered before outpatient CAR T administration through clear checklists, CAR T information packets, and wallet cards post-infusion (28, 32, 34).

Close patient monitoring during treatment administration and throughout the 30-day follow-up period post-infusion is another important aspect of CAR T administration in the outpatient setting. Efficient remote patient monitoring systems to assist patients, caregivers, and medical staff with early identification of AEs may be considered (28). Remote patient monitoring systems, such as in-home Bluetooth and electronic health connected devices are innovative methods for patient support and monitoring, as these devices are capable of monitoring vital signs and temperature changes (31, 37). However, intermittent and missing data may present challenges for their use (31, 37). On the patient side, securing accommodation in close proximity to the outpatient treatment center for at least 4 weeks following infusion in order to meet requirements for close patient monitoring is a challenge and may present a barrier to treatment access (6, 34).

Two additional recommendations to establish a successful outpatient CAR T program identified in the review include the creation of multidisciplinary teams that can efficiently communicate with each other to manage patients throughout the outpatient journey (30, 32, 35), and the development of straightforward patient intake processes encompassing financial eligibility review and provision of patient education materials (28).

### Phase 2: semi-structured exploratory interviews

Two certified CAR T centers were identified for inclusion in Phase 2 (number of experts: Center A: N=2; Center B: N=3) (**Table 2**). Both centers had treated at least 15 patients with commercial cilta-cel for RRMM in the outpatient setting. One interview per center was conducted. Participants included 2 treating clinicians, 2 CAR T nurse coordinators, and 1 director of a CAR T program.

Findings from the semi-structured exploratory interviews revealed that patient selection criteria for outpatient cilta-cel administration were similar across centers, while requirements for post-infusion follow-up, management of AEs, and reimbursement policies differed.

In accordance with Phase 1 findings, participants from both centers emphasized the importance of proper education and training for patients, caregivers, and medical staff, including regular educational updates. Specifically, patients and caregivers should be familiar with the toxicity symptoms related to CAR Ts that may arise and should be able to identify AEs that need to be escalated to their care team for potential transfer to inpatient care. Medical staff should be fully educated and trained in triaging CAR T patients when they call to report AEs. Due to the need for patient monitoring and identification of emerging AEs, participants further highlighted that patients who lack caregiver support are less likely to be considered for outpatient administration than patients who have caregiver support. Therefore, lack of caregiver support was identified as a main challenge for outpatient CAR T administration.

The second key challenge identified that may limit patients’ access to or eligibility for outpatient administration of CAR Ts, including cilta-cel, is the lack of lodging support. CAR T administration centers require patients receiving outpatient treatment to visit the center frequently; patients receiving cilta-cel must visit the center every day for up to 14 days, and 2 or 3 times per week for the following 2 weeks after infusion. Patients are therefore required to seek lodging close to the center (i.e., within 30 minutes travel time) for 30 days post-infusion. Lodging for patients is usually identified with help from the clinical care team and institution, which may be able to provision lodging or has relationships with nearby hotels or facilities. However, the high volume of care provided by larger centers for outpatient services other than CAR T administration may limit the availability of lodging for patients receiving CAR T in some geographical areas. Furthermore, participants noted that many payers may not include or limit coverage for patients’ and caregivers’ lodging and travel costs, as these are not part of the typical treatment reimbursement structure, which often results in additional out-of-pocket expenses for the patient and/or caregiver. Since cilta-cel administration is confidently managed in the outpatient setting, a 14-day duration (versus the standard 28–30 days) for outpatient lodging could be considered for patients who live in close proximity to the center, potentially improving insurance coverage for lodging and lessening potential out-of-pocket expenses.

Furthermore, participants described the importance of having dedicated infrastructure in place to proactively monitor patients who may need to be admitted to the hospital for management of AEs. This infrastructure consideration will likely need to vary between centers as inpatient processes and protocols do not always account for patients transferring from outpatient to inpatient care. These infrastructure considerations include having a dedicated space or beds available in participating centers for outpatient-administered CAR T patients who may need to be directly admitted, an oncology-based urgent care center, dedicated staff ready to care for them, and standard operating procedures (SOPs) for AE management.

### Phase 3: expert panel interviews

Building on Phases 1 and 2, the expert panel interviews brought together 14 participants, including 5 clinicians, 4 administrators, 3 directors, and 2 CAR T nurse coordinators, from 6 centers (Center A: N=3; Center B: N=3; Center C: N=2; Center D: N=2; Center E: N=2; Center F: N=2) to discuss their experiences around outpatient administration of CAR T, with a focus on cilta-cel administration (**Table 2**). Two 90-minute virtual panel discussions took place, where the first expert panel included 7 participants from 3 centers, and the second panel included 7 participants from 4 centers. Participants had experience with administration of CAR T in the outpatient setting across a range of organizations and geographical regions in the US (**Table 2**). Two centers were standalone cancer centers and 3 centers were Prospective Payment Systems (PPS) exempt (**Table 2**). Notably, 4 of the 6 centers had infused cilta-cel in an outpatient setting for at least 15 patients, one of which had infused cilta-cel in over 30 patients in the outpatient setting, while the remaining 2 centers had administered cilta-cel in an outpatient setting to at least 10 patients prior to the expert panels.

All participants (N=14 [100%]) agreed that cilta-cel can be safely and effectively administered in an outpatient setting. Participants from 4 of the 6 centers (67% of participating centers) reported that at the time of the study, their center infused all commercial cilta-cel and ide-cel in the outpatient setting, except in patients with certain comorbidities, such as active neurological complications (e.g., paralysis) or organ dysfunction (e.g., cardiac dysfunction or renal insufficiency needing hemodialysis). The remaining 2 centers (33% of participating centers) reported that they still infused all commercial ide-cel in the inpatient setting in order to closely monitor the patients due to potential early onset of AEs. Infrastructure for outpatient administration, SOPs, AE identification, and patient management (e.g., dedicated teams, 24/7 call center availability, after-hour management, patient lodging arrangements), as described by panel participants, were similar between centers.

**Figure 1:**
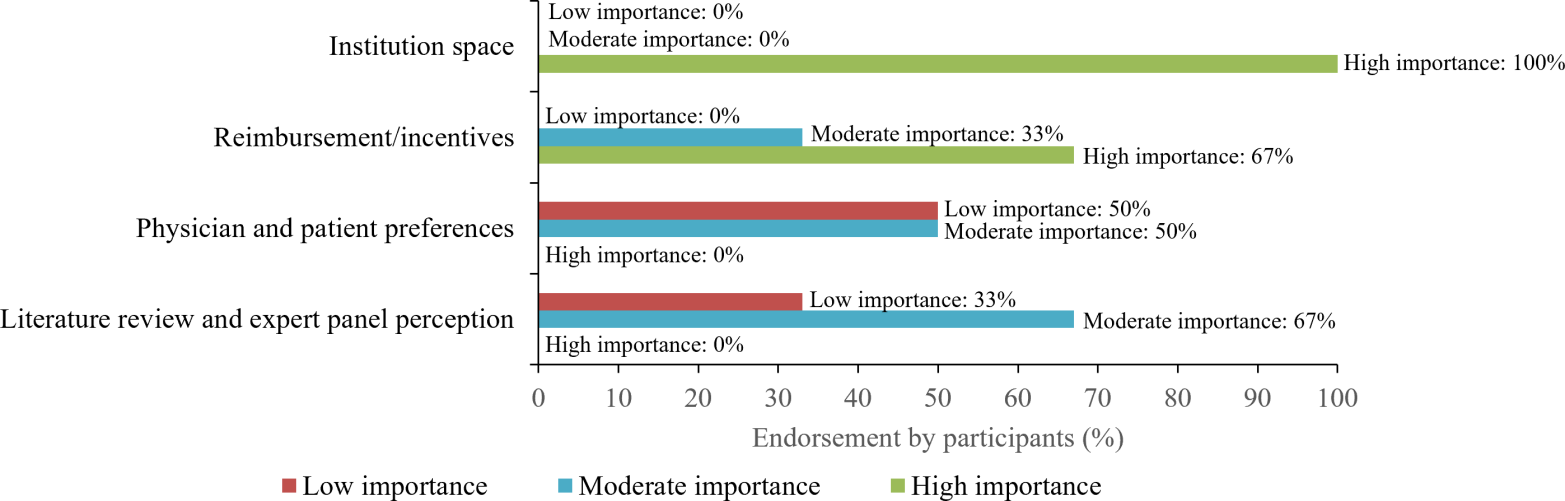
Important factors in the decision-making process for administering CAR T in the outpatient setting. Line coloring corresponds to the proportion of participants that thought each factor was of low (red), moderate (blue), or high (green) importance. CAR T, chimeric antigen receptor T-cell therapy.

When ranking the importance of various aspects of the overall decision-making process for administering CAR Ts in an outpatient setting, all panel participants rated the availability of institutional space to be of high importance and reimbursement or incentives to be the next most important factor (**Figure 1**). Half (50%) of the centers ranked “clinician/patient preference” as moderately important when deciding to treat with CAR T in the outpatient setting, while the remaining half ranked it to be of low importance. Literature review and expert panel perception were considered moderately important when assessing safety and toxicity management in an outpatient setting (**Figure 1**).

A determining factor for many centers to administer cilta-cel in an outpatient setting was the delayed onset of toxicity (e.g., CRS, ICANS) compared with other therapies, such as ide-cel, that have an earlier onset. Participants noted that SOPs are evolving to include management of certain AEs, such as grade 1 CRS, in an outpatient setting. Some of the participating centers administered tocilizumab to treat grade 1 CRS in the outpatient setting. While recommended as an emerging practice in the literature, most centers (5 [83%] participating centers) reported that they do not utilize remote patient monitoring systems for cilta-cel to track AEs due to challenges associated with these systems (e.g., false alarms, anxiety) and resource constraints (e.g., burden to review the data on these devices), relying solely on their robust patient and caregiver education and compliance history for monitoring AEs post-infusion.

Participants from 4 out of the 6 centers noted that payers wanted to be informed if cilta-cel is to be administered in an inpatient or outpatient setting. It is therefore considered best practice by some centers to submit prior authorization applications for both inpatient and outpatient administration simultaneously in case patients need to be admitted for inpatient care after outpatient infusion of the CAR T, as approval times can be long and prior authorizations are valid for 6–12 months once granted.

**Figure 2:**
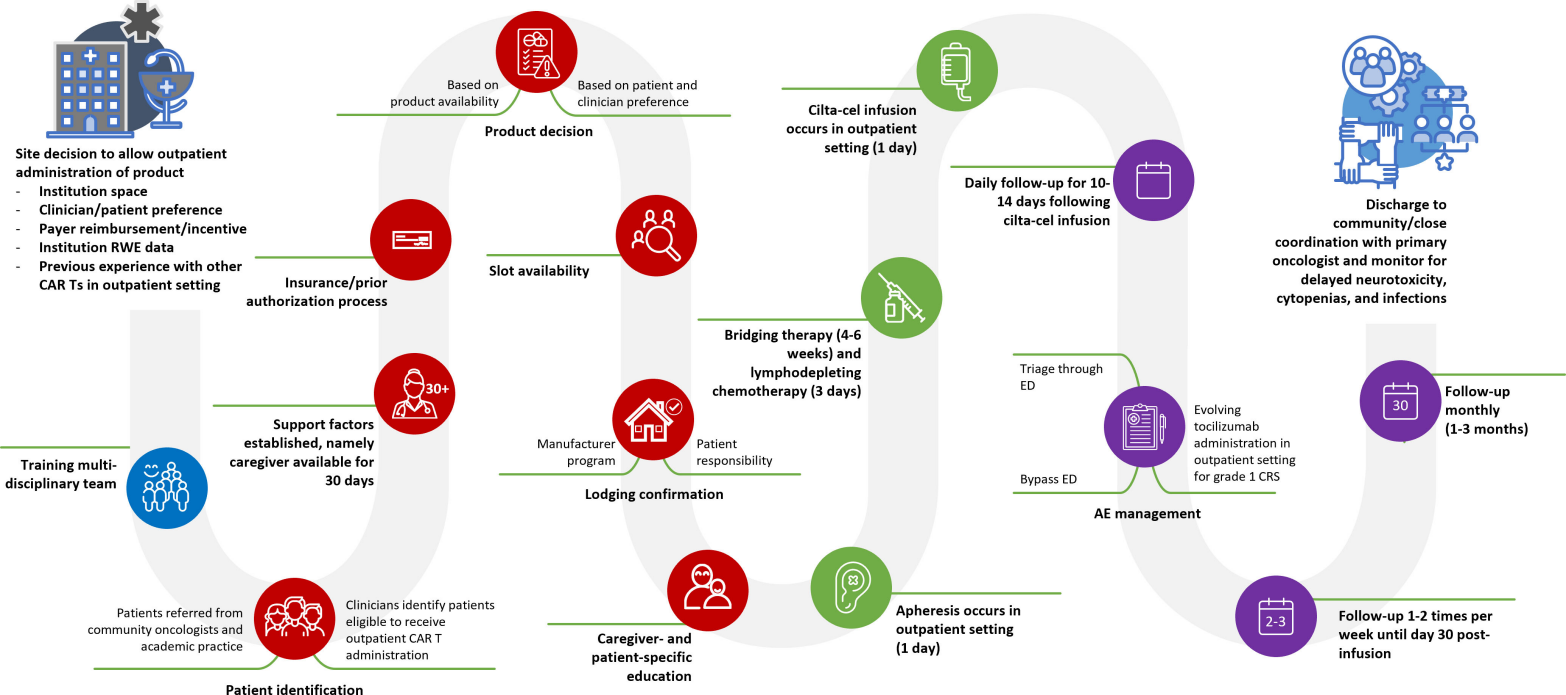
Cilta-cel patient journey as defined by participants of the qualitative study. AE, adverse event; CAR T, chimeric antigen receptor T-cell therapy; cilta-cel, ciltacabtagene autoleucel; CRS, cytokine release syndrome; ED, emergency department; RWE: real-world evidence.

The patient journey map was created based on information provided by participants (**Figure 2**). Patients receiving cilta-cel in the outpatient setting are commonly followed daily for 10-14 days post-infusion, and then once or twice weekly until day 30 post-infusion due to delayed toxicity concerns. After this timeframe, patients are followed up monthly for 1–3 months post-infusion (**Figure 2**). Given evolving concerns regarding delayed neurotoxicity with cilta-cel, participating centers frequently reported follow-up with patients for the first 3-6 months and every 3 months thereafter.

The panel participants provided specific recommendations regarding the infrastructure and SOPs needed for appropriate set-up of an outpatient CAR T center and to safely administer cilta-cel in an outpatient setting (**Table 3**). Recommendations from experts in each panel were similar and consistent with the other phases of the study, such as creation of educational resources for patients and caregivers, creation of SOPs, dedicated outpatient infrastructure and establishment of interdisciplinary teams, outpatient monitoring for toxicity management, and monitoring of the reimbursement landscape (**Table 3**).

**Table 3:** Key recommendations/solutions for successful administration of outpatient CAR T, with a focus on cilta-cel.

Topic	Recommendations
Education	Create educational resources to support the patients and caregivers • To support patients and caregivers through comprehensive education with CAR T information packets, wallet cards post-infusion on symptom monitoring, and identification of factors warranting the need for hospital admission
Standard operating procedures and guidelines	Devise thorough standard operating procedures or product-specific guidelines for outpatient administration of CAR T • To support care teams, it is crucial to have general CAR T protocols or specific outpatient cilta-cel guidelines for 24/7 patient management in place • Staff education regarding recognition, triaging, and management of CAR T related AEs, including information on prophylactic treatment for immune-mediated toxicities
Infrastructure and medical staff	Utilize dedicated outpatient infrastructure • Dedicated facilities available for administration of CAR T with trained staff and available beds • Robust multidisciplinary team for recognizing/managing AEs and infrastructure for transition to inpatient management of severe AEs
Outpatient monitoring	Utilize outpatient monitoring processes for long-term follow-up • Monitor patients for management of delayed neurotoxicity, cytopenias, and infections
Reimbursement	Monitor reimbursement landscape closely • Reimbursement is a key factor for outpatient administration of CAR T • Care team support in identification of payer coverage for lodging and travel costs

AE, adverse event; CAR T, chimeric antigen receptor T-cell therapy; cilta-cel, ciltacabtagene autoleucel.

## Discussion

This mixed methods qualitative study combining a targeted literature review with expert interviews and panels evaluated the clinical and administrative perspectives on commercial cilta-cel outpatient administration from certified CAR T centers in the US. By comprehensively assessing clinical, logistical, and administrative aspects of CAR T administration, this study identified challenges faced by clinicians, administrative staff, patients, and caregivers, as well as recommendations for best practices for outpatient administration of cilta-cel.

CAR T has typically been administered in an inpatient setting due to the risk of serious AEs (such as CRS and ICANS) observed in clinical trials, and with outpatient administration seen as challenging due to the need for close patient monitoring (6, 42). However, cilta-cel has been shown to have a generally predictable toxicity profile in which CRS and ICANs manifest at day 7–8, potentially making it a preferable candidate for outpatient administration (5, 43). The findings of our study highlight key challenges faced by outpatient CAR T centers, notably: limited institutional capacity, the need for multidisciplinary teams, and the need for protocols for CAR T complications. This is supported by 2 recent articles that have highlighted similar findings to the current study with regard to key drivers of outpatient administration of CAR T for hematologic cancers (predictability of AEs, reduced healthcare burden, and patient quality of life), limiting factors (financial and caregiver support), and best practices (dedicated infrastructure, close monitoring of patients, and patient and caregiver education) (24, 42). An expert roundtable also described the importance (in all administration settings) of close patient monitoring, financial and caregiver support considerations, and collaboration across multidisciplinary teams in the administration of CAR Ts for patients with RRMM (44). The current study also highlighted the critical logistical challenges faced by some patients in securing lodging during the 30-day period following outpatient CAR T administration. This aligns with findings from a systematic literature review that identified logistical considerations (e.g., caregiver support, lodging in close proximity to the treatment center) as an important element to consider when administering CAR Ts in an outpatient setting (24).

The key recommendations and solutions for successful administration of outpatient CAR T derived from the current study include the need for education, SOPs and guidelines for monitoring and management of early AEs, dedicated outpatient infrastructure, outpatient monitoring for long-term follow-up, and understanding of the reimbursement landscape (including lodging and travel costs). In recent literature, implementation of these recommendations and solutions has been found to be beneficial. For instance, one study reported on the successful implementation of a clinician education program which addressed knowledge gaps and skills related to outpatient administration of CAR T for the treatment of hematologic cancers (45). In addition, adherence to SOPs and the collaborative use of multidisciplinary teams in the outpatient setting have been underscored across diverse hematologic diseases, including in the Outreach study, which demonstrated successful outpatient infusion and monitoring of CAR T toxicities using SOPs and multidisciplinary teams in large B-cell lymphoma (46, 47). Other studies have emphasized the importance of having a dedicated, developed infrastructure for patients who experience an AE after infusion (28, 32).

Another element that study participants ranked as being of moderate or high importance in the decision to administer cilta-cel in an outpatient setting was reimbursement/incentives. By shifting CAR T administration to an outpatient setting, there is a potential for substantial cost savings and lower healthcare resource utilization, as shown in a systematic review by Hansen et al. reporting 2–4 times higher post-infusion costs and 2–3 times longer length of stays for inpatient relative to outpatient CAR T administration (24). Also, in relapsed/refractory large B-cell lymphoma, a retrospective study by Palomba et al. showed that the post-infusion monitoring costs of outpatient CAR T administration were lower than those of inpatient CAR T administration (48). That study also noted that safety and efficacy outcomes were similar between the inpatient and outpatient settings. Furthermore, Linhares et al. suggested that the reduction in frequency of inpatient stays and length of stays observed among patients receiving lisocabtagene maraleucel (liso-cel) infusion in the outpatient setting may reduce CAR T costs (46).

Overall, a potential impact of the current study is that outpatient CAR T administration is not just about logistical convenience or economic benefit; it is also about reshaping the treatment landscape to create a more sustainable, patient-centered approach that does not just aim for effective therapy but also considers patients’ overall well-being and caregiver support, healthcare resources, and financial efficiency. This study provides additional evidence of general acceptance and agreement among clinical CAR T experts with outpatient experience that cilta-cel can be administered safely in an outpatient setting, as supported by 4 recent studies demonstrating the safety and feasibility of cilta-cel administration in the outpatient setting (14–16, 49). The evidence is grounded in the patient journey map presented in **Figure 2**, which intricately details the sequential steps and decision-making processes integral to outpatient cilta-cel administration. The map shows the complexities of the processes involved throughout the patient journey from the center decision to allow outpatient administration of cilta-cel, all the way to discharge to community practice/close coordination with primary oncologist. Created based on the 3 phases of the current study, the patient journey map encapsulates diverse perspectives and shows a holistic approach of the best practices for optimizing the patient journey for outpatient cilta-cel administration, and also leaves room for improvement as more real-world evidence is published. Moreover, the initial utilization of mixed methods to evaluate best practices, as demonstrated in the current study by combining literature review and expert panels, may facilitate earlier implementation of outpatient infusion services across a broader range of locations, including centers with more limited resources. This approach can leverage insights from larger institutions, fostering greater confidence among individual facilities. To complement our study, long-term real-world evidence of clinical outcomes of outpatient cilta-cel administration is needed.

### Strengths and limitations

This mixed methods study exhibits several strengths, due to its comprehensive methodology. By integrating a targeted literature review, semi-structured interviews, and expert panels, it provided a holistic understanding of outpatient cilta-cel administration. Using a combination of the above methods helped overcome the limitations associated with each of them individually. For instance, conducting a literature review will capture all published recommendations; however, it may not always represent the most current guidelines in a rapidly evolving disease space. On the other hand, expert panels will provide important up-to-date and practical insights, but results may only be representative of the experts’ respective experiences. By combining a targeted literature review with expert panel discussions informed by the available literature, the current study provides a balanced incorporation of empirical evidence with practical insights guiding the current state of the practice. The inclusion of diverse experts from various roles and geographical locations ensured a multifaceted perspective, validating the findings from the targeted literature review and enriching the study with real-world insights. Furthermore, information gathered from this study could be practice-informing and greatly benefit centers that may have limited resources for conducting such assessments.

However, there were also some limitations. The scarcity of literature specifically addressing outpatient CAR T for RRMM posed a challenge, potentially limiting the depth of background information. Additionally, while the study included diverse centers, particularly those with high outpatient volumes, the sample size might limit the complete representation of all CAR T centers (e.g., community-based centers), impacting the generalizability of our findings. There is a need for larger studies and bodies of evidence from a diverse representation of centers. In addition, differences in SOPs may exist between centers (e.g., depending on their location), but could not be assessed as part of the current study. Lastly, this study’s focus on cilta-cel might affect the broader applicability of its conclusions to other CAR Ts or hematologic malignancies.

## Conclusion

This study offers a comprehensive understanding of the feasibility of outpatient cilta-cel administration in participating CAR T centers, delineating actionable recommendations and acknowledging existing challenges. Key factors associated with successful outpatient CAR T administration include the availability of dedicated outpatient infrastructure, education and training for patients and caregivers, as well as SOPs for multidisciplinary care teams, including outpatient monitoring processes for long-term follow-up as well as payer reimbursement. Best practices for outpatient management and follow-up are evolving, which underscores the importance of ongoing research to guide centers in implementing effective outpatient administration of CAR Ts.

## Data availability statement

The original contributions presented in the study are included in the article/**Supplementary Materials**. Further inquiries can be directed to the corresponding author.

## Ethics statement

The requirement of ethical approval was waived by WCG Ethical and Scientific Review for Clinical Studies for the studies involving human subjects. The studies were conducted in accordance with the local legislation and institutional requirements. The participants provided their written informed consent to participate in this study.

## Author contributions

DH: Writing – original draft, Writing – review & editing, Supervision. BD: Writing – original draft, Writing – review & editing. MH: Writing – original draft, Writing – review & editing. DD: Writing – original draft, Writing – review & editing. TJ: Writing – original draft, Writing – review & editing. CH: Writing – original draft, Writing – review & editing. MJ: Writing – original draft, Writing – review & editing. Y-HL: Writing – original draft, Writing – review & editing, Conceptualization, Methodology, Supervision. KD: Writing – original draft, Writing – review & editing. NL: Writing – original draft, Writing – review & editing, Conceptualization, Investigation, Methodology, Project administration. JS: Writing – original draft, Writing – review & editing, Conceptualization, Investigation, Methodology, Project administration, Visualization. PO: Conceptualization, Investigation, Methodology, Project administration, Visualization, Writing – original draft, Writing – review & editing. LM: Writing – original draft, Writing – review & editing. MP: Writing – original draft, Writing – review & editing. SH: Writing – original draft, Writing – review & editing, Conceptualization, Methodology, Supervision. ZQ: Supervision, Writing – original draft, Writing – review & editing, Project administration. KP: Writing – original draft, Writing – review & editing.

## Supplementary material

Supplementary Table 1 Overview of pre-read contents and objectives (Phase 2).
