# Supplementary material for: Clinician and administrator perspectives on outpatient administration of ciltacabtagene autoleucel in relapsed or refractory multiple myeloma
Source: Front Immunol. 2024 Jun 10;15:1405452. doi: 10.3389/fimmu.2024.1405452 (PMC11194690; doi:10.3389/fimmu.2024.1405452)
Supplement: Supplementary Table 1 — Overview of pre-read contents and objectives (Phase 2). [file Table_1.docx]

Supplementary Material

Clinician and Administrator Perspectives on Real-world Experience with Outpatient Administration of Ciltacabtagene Autoleucel Chimeric Antigen Receptor T-Cell Therapy in Relapsed or Refractory Multiple Myeloma

Doris K. Hansen MD^*^, Binod Dhakal MD, Mehdi Hamadani MD, David Dingli MD PhD, Tania Jain MD, Carol Ann Huff MD, Murali Janakiram MD, Yi-Hsuan Liu PhD MS RD, Kevin C. De Braganca, Nicole Lodowski MPH, Jennifer Sander MPH, Peter Okorozo PharmD, Lindsay McFarland MN RN OCN, Matthew Perciavalle PharmD, Stephen Huo PhD MD MSPH, Zaina P. Qureshi PhD, MS, MPH, and Krina K. Patel MD Msc

***Correspondence:** Doris K Hansen, MD. [Doris.Hansen@moffitt.org](mailto:Doris.Hansen@moffitt.org)

# Supplementary Tables

**Supplementary Table 1. Overview of pre-read contents and objectives (Phase 2)**

| Key sections of the pre-read | Objectives |
| --- | --- |
| Interview discussion topics | To present the flow of discussion. |
| CAR T patient journey | To explore the patient journey and understand key differences, best practices, and challenges between centers, including ways to improve patient experience. |
| CAR T outpatient center setup and processes | To discuss best practices and challenges during initial center set-up, including understanding multidisciplinary team set ups between outpatient and hospital practices, and the key processes involved for setting up an outpatient CAR T center. |
| Patient identification and pre-treatment coordination | To discuss best practices and challenges during early stages of patient identification and coordination, including understanding patient identification and evaluation processes for outpatient CAR T administration, and the coordination required in this process. |
| Treatment administration and patient monitoring | To discuss best practices and challenges of outpatient CAR-T administration and monitoring including management and monitoring of adverse events such as delayed CRS and neurologic toxicities in the outpatient setting. |
| Overall best practices | To discuss any other topics not addressed, and understand the major clinical, financial, and administrative challenges at the participants’ respective outpatient CAR T center. |
| Background research findings | To present the overview of outpatient CAR T administration in the US. |

CAR T: chimeric antigen receptor T-cell therapy; CRS: cytokine release syndrome; US: United States.
